# Molecular Characteristics of Disease-Causing and Commensal *Staphylococcus lugdunensis* Isolates from 2003 to 2013 at a Tertiary Hospital in Taiwan

**DOI:** 10.1371/journal.pone.0134859

**Published:** 2015-08-06

**Authors:** Chun-Fu Yeh, Tsui-Ping Liu, Chun-Wen Cheng, Shih-Cheng Chang, Ming-Hsun Lee, Jang-Jih Lu

**Affiliations:** 1 Division of Infectious Diseases, Department of Internal Medicine, Chang Gung Memorial Hospital at Linkou, Chang Gung University College of Medicine, Taoyuan, Taiwan; 2 Graduate Institute of Clinical Medical Sciences, College of Medicine, Chang Gung University, Taoyuan, Taiwan; 3 Department of Laboratory Medicine, Chang Gung Memorial Hospital at Linkou, Taoyuan, Taiwan; 4 Department of Medical Biotechnology and Laboratory Science, College of Medicine, Chang Gung University, Taoyuan, Taiwan; Faculdade de Medicina de Lisboa, PORTUGAL

## Abstract

**Objectives:**

*Staphylococcus lugdunensis* can cause community- and healthcare-associated infections. This study investigated the molecular characteristics of *S*. *lugdunensis* isolates collected at our hospital and compared the characteristics of the infectious and commensal isolates.

**Methods:**

We collected the *S*. *lugdunensis* isolates between 2003 and 2013. The antimicrobial resistance test, SCCmec typing, accessory gene regulator (*agr*) typing, pulsed-field gel electrophoresis (PFGE), and δ-like hemolysin activity were performed.

**Results:**

In total, 118 *S*. *lugdunensis* isolates were collected, of which 67 (56.8%) were classified into the infection group and 51 (43.2%) into the commensal group. The oxacillin resistance rate was 36.4%. The most common SCCmec types were SCCmec types V (51.4%) and II (32.6%). In total, 34 pulsotypes were identified. The PFGE typing revealed five clones (pulsotypes A, J, M, N, and P) at our hospital. Pulsotypes A and N caused the spread of high oxacillin resistance. In total, 10.2% (12 of 118) of the isolates lacked δ-like hemolysin activity. Compared with the infection group, the commensal group showed a higher percentage of multiple drug resistance and carried a higher percentage of SCCmec type II (11 of 22, 50% and 3 of 21, 14.3%) and a lower percentage of SCCmec type V (8 of 22, 36.4% and 14 of 21, 66.7%). The commensal group (27 PFGE types) showed higher genetic diversity than did the infection group (20 PFGE types). No difference was observed in the distribution of the five main pulsotypes, *agr* typing, and the presence of δ-like hemolysin activity between the two groups.

**Conclusions:**

Five main clones were identified at our hospital. The commensal group showed higher genetic diversity, had a higher percentage of multidrug resistance, and carried a higher percentage of SCCmec type II and a lower percentage of SCCmec type V than did the infection group.

## Introduction

*Staphylococcus lugdunensis*, belonging to the group of coagulase-negative staphylococci (CoNS), was first reported by Freney et al. in 1988 [1]. Although the incidence rate of *S*. *lugdunensis* infection was low [2, 3], increasing numbers of patients with *S*. *lugdunensis* infective endocarditis have been reported in recent 20 years [4, 5]. It has also emerged as a pathogen that causes various community- and healthcare-associated infections, such as those of the bloodstream, bones and joints, skin and soft tissues, and the central nervous system [6, 7].

Like other CoNS species, *S*. *lugdunensis* is a commensal skin flora of humans [8]. It can be transmitted between hospitalized patients and hospital environments and causes invasive infections in patients with impaired skin integrity and indwelling catheters and foreign devices [9, 10]. The organism is generally assumed to colonize the human skin and then cause invasive infection. However, little is known about the molecular epidemiology of commensal isolates.

Compared with the high prevalence of oxacillin resistance among *Staphylococcus aureus* and other CoNS in hospital environments, that of *S*. *lugdunensis* has been reported to be low [3, 6, 9, 11, 12]. However, in our recent study, a high rate of oxacillin resistance was observed in patients with invasive *S*. *lugdunensis* infection, with a 20.8% resistance rate [7]. In addition, erythromycin (25%) and clindamycin (18.8%) resistance was observed. The most common SCCmec type was type V, and all these isolates were accounted for healthcare-associated infections [7]. *S*. *lugdunensis* isolates carrying SCCmec type V were also reported in central Taiwan [13].

In addition to SCCmec typing, the accessory gene regulator (*agr*) locus also serves as a crucial molecular marker of staphylococci. According to polymorphisms, the *agr* gene can be divided into four types in *S*. *aureus* [14], and two types in *S*. *lugdunensis* [15]. Many virulence factors of staphylococci are regulated by the *agr* locus. Extensive research has been conducted on the *agr* locus of *S*. *aureus*, and different *agr* types have been associated with different virulence profiles and human diseases [16]; however, the association between *agr* typing and *S*. *lugdunensis* infection is unclear. Like *S*. *aureus* and *S*. *epidermidis*, *S*. *lugdunensis* can produce *S*. *lugdunensis* synergistic hemolysin, which belongs to the family of phenol-soluble modulin (PSM) peptides [17]. This hemolysin is controlled by the *agr* locus. This δ-like hemolysin produced serves as a surrogate maker of the *agr* function in staphylococci [18].

Although the molecular characteristics of pathogenic isolates have been studied [19, 20], little is known about the characteristics of commensal populations. Understanding the molecular characteristics of infectious and commensal isolates can facilitate identifying the cause of the high resistance rate, control the spread of resistant strains, and establish a background database for further study on pathogenic isolates. The aim of this study was to investigate the molecular characteristics of the *S*. *lugdunensis* isolates collected at our hospital over 10 years and to compare the characteristics of the isolates causing infection and those considered contaminants or commensals by using various methods, including pulsed-field gel electrophoresis (PFGE) typing, SCCmec typing, *agr* typing, and hemolytic activity analysis.

## Materials and Methods

### Clinical setting

This study was conducted at Chang Gung Memorial Hospital (CGMH) in Taoyuan, Taiwan. The hospital is a 3700-bed university-affiliated hospital and tertiary referral medical center in northern Taiwan. This study was approved by the Chang Gung Medical Foundation Institutional Research Board (approval number: 103-3231B). All *S*. *lugdunensis* isolates were obtained from blood or sterile body fluid cultures in our clinical microbiology laboratory between May 2003 and 2013. Clinical data, including the patient age, sex, types of samples, and infectious foci, were retrospectively collected using chart review and analyzed anonymously.

### Case definition

Clinically significant bacteremia was defined as occurring in patients when two consecutive positive blood cultures were obtained for *S*. *lugdunensis*. Patients with a single positive blood culture were considered to have clinically significant bacteremia if they experienced one or more of the following: prolonged fever, hypotension, leukocytosis or neutropenia with a left-shifted differential, or disseminated intravascular coagulopathy combined with risk factors for infections caused by skin flora, including long-term intravascular catheterization, peritoneal dialysis or hemodialysis, or extensive postsurgical infections with CoNS [21, 22]; otherwise, positive results were attributed to contamination of the blood culture, and the isolate was classified as commensal.

Healthcare-associated infections were defined as occurring in patients with the following: (1) *S*. *lugdunensis* infection identified after 48 hours of admission to the hospital; (2) a history of hospitalization, surgery, dialysis, or residence in a long-term care facility within 1 year of the positive culture date; or (3) a permanent indwelling catheter or percutaneous medical device present at the time of culture. Cases that had none of the above features were classified as community associated [23].

### Microbiological methods

All *S*. *lugdunensis* isolates were first identified using Gram staining and biochemical methods (catalase positive, coagulase negative, pyrrolidonyl arylamidase positive, and ornithine decarboxylase positive). Furthermore, all isolates were verified using the polymerase chain reaction (PCR) method described by Noguchi et al. [24] and a Bruker Biotyper (database 2.0) matrix-assisted laser desorption ionization/time of flight mass spectrometry system [25].

### Antimicrobial susceptibility testing

The minimal inhibitory concentration (MIC) of oxacillin was determined using agar dilution methods. The oxacillin resistance was defined as an MIC of 4 mg/mL or greater, according to the Clinical and Laboratory Standards Institute guidelines [26]. Susceptibility testing of penicillin, clindamycin, erythromycin, and trimethoprim–sulfamethoxazole was performed using the disk diffusion method and interpreted according to the Clinical and Laboratory Standards Institute guidelines [26].

### SCCmec typing

SCCmec typing of the *S*. *lugdunensis* isolates was performed using a multiplex PCR for identifying the *ccr* gene complex, the *mec* gene complex, and specific structures in the adjacent regions as described previously when the oxacillin MIC was ≥4 μg/mL [27].

### *agr* typing

**Fig 1:**
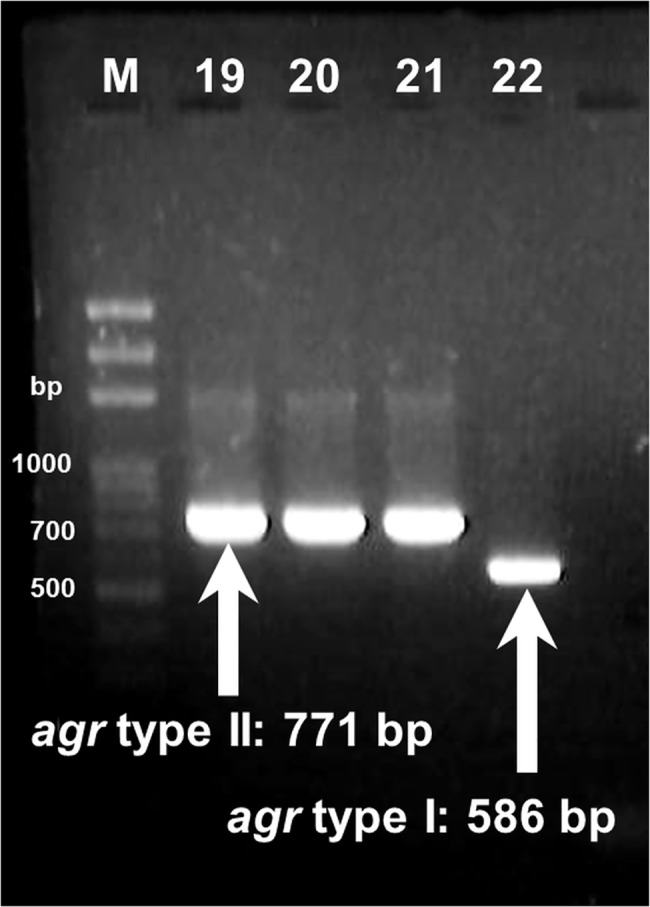
Multiplex PCR for agr gene typing of *Staphylococcus lugdunensis*. (Lanes M: marker; 19: *S*. *lugdunensis* No. 19; 20: *S*. *lugdunensis* No. 20; 21: *S*. *lugdunensis* No. 21; 22: *S*. *lugdunensis* No. 22).

Two PCR primers (SL_agr-F and SL_agr-R) were designed to detect the *agr* genes of the *S*. *lugdunensis* isolates. We then determined the *agr* type through PCR amplification of the conserved sequences from the *agrC* gene, with two forward primers and one reverse primer (SL_agr-1-F, SL_agr-2-F/SL_agr-R). All primers were designed using a computer-assisted analysis of the genomic DNA sequences from GenBank accession numbers AF173933 and AF346728 and the whole genome sequence data of our two *S*. *lugdunensis* isolates. All primer sequences are listed in Table 1.The *agr* specificity types were identified on the basis of the expected product sizes (586 bp for agr type I and 771 bp for agr type II) (Fig 1).

**Table 1:** Primers used in polymerase chain reaction amplification and sequencing of the *agr* locus in this study.

Primer	5′-3′ Sequence	Gene	Purpose	Size of amplicon (bp)
SL_agr-F/SL_agr-R	ATAATGATACCAAGGAGCGTG/ CGAACCTTTAGCTTATCTGTACC	*agrB*/*agrC*	screening	1626
SL_agr-1-F/SL_agr-R	CTGTCATCCTTAGTGTAATTGCTG/ CGAACCTTTAGCTTATCTGTACC	*agrC*	*agr* I typing	586
SL_agr-2-F/SL_agr-R	GCCGGCATAATAGTCCCTTCTG/CGAACCTTTAGCTTATCTGTACC	*agrC*	*agr* II typing	771

### Pulsed-field gel electrophoresis

Genomic DNAs of the *S*. *lugdunensis* isolates were digested with SmaI and separated using PFGE as described previously [28]. Lambda ladder DNA was used as a molecular weight marker for PFGE. The PFGE patterns were analyzed, and a dendrogram was constructed using BioNumerics software 6.0 (Applied Maths; Texas, USA). Percent similarities were identified on a dendrogram derived using the unweighted pair group method by using arithmetic averages and based on Dice coefficients. Band position tolerance and optimization were set at 1.25 and 0.5%, respectively [28]. A similarity coefficient of 80% was selected to define closely related strains (clones).

### Hemolytic activity

The δ-like hemolysin activity was analyzed by performing a cross-streaking test, perpendicular to RN4220, which produces only β-hemolysin on a Columbia blood agar plate. We used ATCC 25923 *S*. *aureus* as the *agr*-positive control and the *S*. *aureus* strain Mu 50 as the *agr*-negative control.

### Statistical analysis

Statistical analyses were performed using the Statistical Package for Social Sciences software Version 18.0 (SPSS Inc.; Chicago, IL, USA). The frequency between the two groups was analyzed by performing a z-test.

## Results

In total, 118 *S*. *lugdunensis* isolates were collected from 118 patients at CGMH. The mean age of patients was 56.0 ± 28.0 years, and 61 patients (51.7%) were male. All isolates tested were confirmed using matrix-assisted laser desorption ionization/time of flight mass spectrometry and PCR methods. Most of the isolates were collected from the blood culture (n = 105, 89%) (Table 2). In total, 67 isolates (56.8%) caused infections, and 51 isolates (43.2%) were considered contaminants and classified as commensals. Among the isolates involved in infection, 59 (50.0%) caused healthcare-associated infections and 8 (6.8%) caused community-associated infections. The leading causes of infection were primary (n = 29, 24.6%) and catheter-related bacteremia (n = 6, 8.5%). The foci of infection are shown in Table 3.

**Table 2:** 118 *Staphylococcus lugdunensis* isolates obtained from various samples.

Sample	Number	Percentage (%)
Blood	105	89.0
Ascites	6	5.1
Body fluid	2	1.7
Synovial fluid	2	1.7
Amniotic fluid	1	0.8
Cerebrospinal fluid	1	0.8
Pleural effusion	1	0.8

**Table 3:** Distribution of infection group and commensal group of 118 *Staphylococcus lugdunensis* isolates.

	Number	Percentage (%)
**Infection group**	67	56.8
Primary bacteremia	29	24.6
Catheter-related	10	8.5
Arteriovenous graft/fistula	6	5.1
Bone and joints	6	5.1
Infective endocarditis	4	3.4
Intra-abdomen	4	3.4
Skin and soft tissue	3	2.5
CAPD peritonitis	2	1.7
Central nervous system	1	0.8
Genital system	1	0.8
Lung	1	0.8
**Commensal group**	51	43.2

### Antimicrobial susceptibility

The results regarding antibiotic resistance are shown in Table 4. Analysis of the 118 *S*. *lugdunensis* isolates revealed that 90 (76.3%) were resistant to penicillin, 43 (36.4%) to oxacillin, 40 (33.9%) to erythromycin, 33 (28.0%) to clindamycin, and only 2 (1.7%) to trimethoprim–sulfamethoxazole. All oxacillin-resistant *S*. *lugdunensis* isolates emerged after the year 2009 and were distributed as follows: 4 of 9 isolates (44%) in 2009, 12 of 30 (40%) in 2010, 11 of 29 (37.9%) in 2011, 11 of 22 (50%) in 2012, and 5 of 9 (35.7%) in 2013. Among the 43 oxacillin-resistant isolates, 18 (41.9%) were also resistant to clindamycin and erythromycin and one (0.8%) was also resistant to erythromycin and trimethoprim–sulfamethoxazole. Compared with the infectious isolates, the commensal isolates showed a higher percentage of multiple drug resistance (*p* < 0.05). No difference was observed in the penicillin and oxacillin resistance rates.

**Table 4:** Antibiotic resistance, distribution of SCCmec types and *agr* types, and δ-hemolytic activity among infectious and commensal *Staphylococcus lugdunensis* isolates.

	Total, n = 118, Number (%)	Infection, n = 67, Number (%)	Commensal, n = 51, Number (%)	*p* value
**Antibiotic resistance**				
Penicillin-R	90 (76.3)	48 (71.6)	42 (82.4)	0.1773
Oxacillin-R	43 (36.4)	21 (31.3)	22 (43.1)	0.187
SCCmec type II^1^	14/43 (32.6)	3/21 (14.3)	11/22 (50.0)	0.012
SCCmec type IV	2/43 (4.7)	1/21 (4.8)	1/22 (4.5)	0.976
SCCmec type V	22/43 (51.2)	14/21 (66.7)	8/22 (36.4)	0.047
SCCmec type Vt	3/43 (7.0)	2/21 (9.5)	1/22 (4.5)	0.522
SCCmec type NT	2/43 (4.7)	1/21 (4.8)	1/22 (4.5)	
Clindamycin-R	33 (28.0)	13 (19.4)	20 (39.2)	0.017
Erythromycin-R	40 (33.9)	17 (25.4)	23 (45.1)	0.025
TMP–SMX-R	2 (1.7)	1 (1.5)	1 (2.0)	0.841
***agr* type**				
I	53 (44.9)	27 (40.3)	26 (51.0)	0.246
II	65 (55.1)	40 (59.7)	25 (49.0)	0.246
**δ-hemolysin activity**				
nonhemolytic	12(10.2)	6 (9.0)	6 (11.8)	0.617
**Main pulsotypes**				
A	25 (21.2)	17 (25.4)	8 (15.7)	0.201
J	14 (11.9)	10 (14.9)	4 (7.8)	0.238
M	8 (6.8)	5 (7.5)	3 (5.9)	0.728
N	12 (10.2)	5 (7.5)	7 (13.7)	0.262
P	7 (5.9)	4 (6.0)	3 (5.9)	0.984

NT = nontypeable; R = resistant; TMP–SMX = trimethoprim–sulfamethoxazole

^1^ The total number of SCCmec types was the number of isolates with oxacillin resistance.

### SCCmec typing

The SCCmec typing results are summarized in Table 4. Among the 43 oxacillin-resistant isolates, SCCmec could be classified in 41 isolates, and 2 isolates were untypeable. The most common SCCmec type was type V (22 of 43, 51.2%), followed by type II (14 of 43, 32.6%). A higher frequency of SCCmec type V (66.7%, 14 of 21) than that of SCCmec type II (14.2%, 3 of 21) was observed in patients with infections (n = 21) caused by oxacillin-resistant *S*. *lugdunensis*. All infectious oxacillin-resistant isolates were isolated from patients with healthcare-associated infections. Among the 22 commensal isolates, SCCmec type II (11of 22, 50%) was the most frequently detected, followed by SCCmec type V (8 of 22, 36.4%). The difference in the distribution of SCCmec types was statistically significant between the infection and commensal groups (*p* < 0.05) (Table 4). All *S*. *lugdunensis* isolates with SCCmec type II were resistant to oxacillin, erythromycin, and clindamycin. Most SCCmec type V isolates were resistant to oxacillin and sensitive to clindamycin and erythromycin (21 of 22, 95.2%).

### *agr* typing

The *agr* gene in most isolates could be divided into two *agr* types according to the PCR product size. Large PCR products were observed in our two isolates (SL32 and SL44). Further analysis of the PCR products using sequencing suggested an insertion sequence at the *agr* locus. The sequencing results showed that SL32 possessed *agr* type I, and SL44 possessed type II. In total, 53 isolates (44.9%) possessed *agr* type I, and 65 (55.1%) possessed *agr* type II. The distribution of the infectious isolates was as follows: 27 isolates (40.3%) carried type I, and 40 isolates (59.7%) carried type II. The distribution of the commensal isolates was as follows: 26 isolates (51.0%) possessed type I, and 25 isolates (49.0%) possessed type II. No statistical difference was observed between the infection and commensal groups (Table 4). In addition, most isolates with SCCmec type II carried *agr* type I (13 of 14, 92.9%), and all isolates with SCCmec type V carried *agr* type II (22 of 22, 100.0%) (*p* < 0.05). The results showed a strong relationship between *agr* and SCCmec types in oxacillin-resistant isolates.

### Hemolytic activity

Upon screening for δ-like hemolysin activity, we found that 12 (10.2%) of the 118 isolates lacked synergistic hemolytic activity; of these isolates, six belonged to the infection group, and six belonged to the commensal group. The percentage of nonhemolytic isolates was similar between the infection and commensal groups (Table 4). Among the nonhemolytic isolates, three isolates (3 of 53, 5.7%) possessed *agr* type I, and nine isolates (9 of 65, 13.8%) possessed *agr* type II. The percentage of nonhemolytic isolates with *agr* types I and II was similar (*p* = 0.144).

### Pulsed-field gel electrophoresis typing

**Fig 2:**
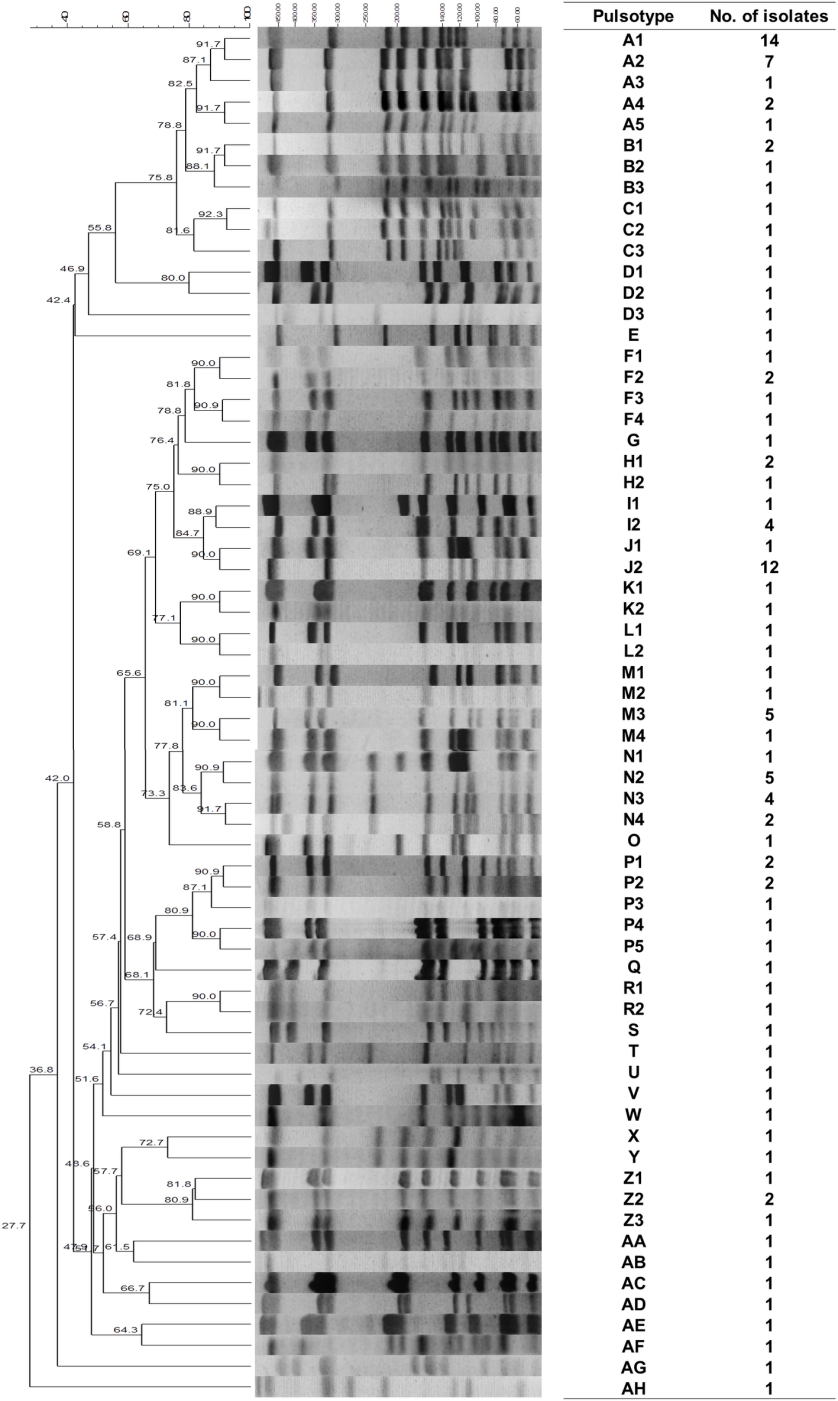
Dendrogram of pulsed field gel electrophoresis of 118 Staphylococcus lugdunensis isolates.

The dendrogram is shown in Fig 2. The 118 *S*. *lugdunensis* isolates collected at our hospital comprised a total of 34 PFGE types; however, more than half of the isolates (55.9%) belonged to five main pulsotypes (A, J, M, N, and P). Nineteen pulsotypes were represented by a single isolate each. Overall, pulsotype A (25/118, 21,2%) was the predominant pulsotype, followed by pulsotype J (14/118, 11.9%), pulsotype N (12/118, 10.2%), pulsotype M (8/118, 6.8%), and pulsotype P (7/118, 5.9%). The five pulsotypes differed in their resistant patterns. More than half of the isolates (n = 21, 84.0%) of pulsotype A were resistant only to oxacillin and carried SCCmec type V–*agr* type II (17/25, 68.0%), while most isolates (n = 11, 91.7%) belonging to pulsotype N showed multidrug resistance and carried SCCmec type II–*agr* type I (9/12, 75.0%) (Table 5). Compared with isolates of pulsotypes A and N, those belonging to pulsotypes J, M, and P were mostly oxacillin sensitive (25/29, 86.2%). Compared with the infection group (20 PFGE types), the commensal group (27 PFGE types) showed higher genetic diversity (*p* = 0.011). No difference was observed in the distribution of the five main pulsotypes between the infection and commensal groups (Table 4). The details of the characteristics of the five pulsotypes are shown in Table 5 and S1 Table.

**Table 5:** Summary of the pulsed-field gel electrophoresis results, antimicrobial resistance, and SCCmec and *agr* typing for *Staphylococcus lugdunensis*.

PFGE type	Number (%) of isolates	Infection, n	Oxacillin resistant, n	Resistance profile ^1^, n	SCCmec-*agr*, n
A	25 (21.2)	HAI, 17; Commensals, 8	21	O, 19; O-C-E, 2	SCCmec V-*agr* II, 17; SCCmec Vt-*agr* II, 3; SCCmec IV-*agr* II, 1
J	14 (11.9)	HAI, 10; Commensals, 4	1	O-C-E, 1; C-E, 4	SCCmec II-agr I, 1
M	8 (6.8)	HAI, 5; Commensals, 3	2	O-C-E, 2; C-E,1	SCCmec II-*agr* I, 2
N	12 (10.2)	HAI, 4; CAI, 1; Commensals, 7	11	O-C-E, 11	SCCmec II-*agr* I, 9; SCCmec II-*agr* II, 1; SCCmec NT-*agr* I, 1
P	7 (5.9)	HAI, 3; CAI, 1; Commensals, 3	1	O-E-SXT, 1; C-E, 2; E, 2; E-SXT, 1	SCCmec IV-agr I, 1
other	52 (44.1)	HAI, 20; CAI, 6; Commensals, 26	7	O, 5; O-C-E, 2; C-E, 8; E, 3	SCCmec V-*agr* II, 5; SCCmec II-*agr* I, 1; SCCmec NT-*agr* I, 1

CAI = community-associated infection; HAI = healthcare-associated infection; NT = nontypeable; PFGE = pulsed-field gel electrophoresis

^1^C-E, clindamycin and erythromycin resistance; E, erythromycin resistance; E-SXT, erythromycin and trimethoprim–sulfamethoxazole resistance; O, oxacillin resistance; O-C-E, oxacillin, clindamycin, and erythromycin resistance; O-E-SXT, oxacillin, erythromycin, and trimethoprim–sulfamethoxazole resistance

## Discussion

In this study, we analyzed general molecular characteristics and compared the molecular characteristics of the *S*. *lugdunensis* isolates at our hospital in the infection and commensal groups. The two most common SCCmec types carried by oxacillin-resistant *S*. *lugdunensis* at our hospital were types II and V. Both *agr* types I and II were equally distributed in the studied *S*. *lugdunensis* isolates. In addition, we identified five main clones at our hospital. Pulsotypes A and N were responsible for high oxacillin resistance. A comparison of the characteristics of the infectious *S*. *lugdunensis* and commensal isolates revealed that the commensal group had a higher percentage of multiple drug resistance than did the infection group. The commensal group also showed higher genetic diversity than did the infection group. The isolates belonging to the commensal group carried SCCmec type II more frequently and SCCmec type V less frequently compared with those belonging to the infection group. No difference was observed in the distribution of *agr* types and in the presence of hemolysin activity between the two groups.

SCCmec typing is a crucial epidemiological technique for analyzing oxacillin-resistant staphylococci. Studies showed that healthcare-associated methicillin-resistant *S*. *aureus* (MRSA) infections were caused by strains carrying SCCmec types I, II, and III but rarely by those carrying types IV and V [29]. At our hospital, the most common oxacillin-resistant *S*. *lugdunensis* isolates causing healthcare-associated infections carried SCCmec type V, followed by SCCmec type II. However, among the isolates considered commensals, the isolates carrying SCCmec type II were more common than those carrying SCCmec type V. Keito et al. in 2011 reporting a gene named *psm-mec*, which was present in the SCCmec type II cassette, regulated the virulence of *S*. *aureus*. The *psm-mec* encoded RNA and peptides could promote biofilm formation and reduced the dissemination ability of staphylococci [30, 31]. The SCCmec type V isolates, which did not carry *psm-mec*, showed higher colony spreading ability. This may partially explain our finding that the isolates carrying SCCmec type II caused fewer infection episodes than did those carrying SCCmec type V. Many studies on the epidemiology of MRSA infection have revealed that the community-associated MRSA SCCmec types have infiltrated healthcare settings [32]. More recent studies have shown the presence of MRSA strains carrying SCCmec types IV and V in healthcare-associated settings in many countries [33–35]. An increase in the number of MRSA strains carrying SCCmec type V was not only associated with skin and soft tissue infections but also with invasive infections. Our study showed that both SCCmec types II and V are the major SCCmec types in healthcare settings. Recently, *S*. *lugdunensis* carrying SCCmec type V was identified in central Taiwan [13]. Our data also suggested the emergence of SCCmec type V in healthcare settings for *S*. *lugdunensis*. However, the number of cases with community-associated infections was small in our study, and further analysis of community-associated SCCmec types in *S*. *lugdunensis* is required.

In the present study, the oxacillin resistance rate was 36.4%, which was higher than that reported in other studies [9, 12]. All oxacillin-resistant isolates were found after 2009. All infection episodes caused by oxacillin-resistant isolates were healthcare-associated infections. No community-associated infection with oxacillin-resistant *S*. *lugdunensis* was found. The findings indicate that the emergence of high oxacillin resistance within *S*. *lugdunensis* was due to high antibiotic selection pressure at the hospital. Moreover, according the PFGE data, 35 (81.3%) oxacillin-resistant isolates belonged to two clones (pulsotypes A and N). These findings suggest that the increasing resistance rate can be largely attributed to clonal spread at our hospital and not to the horizontal transfer of the SCCmec cassette between the *S*. *lugdunensis* isolates. These groups of isolates could not only colonize the skin but also caused invasive infections. While these bacterial clones did not cause outbreaks, they can still cause invasive infections, especially in patients with indwelling catheters. Although we did not screen for bacteria isolated from the hospital environment and medical devices, we believe that infection control precautions are required to eliminate the bacteria.

No difference was observed in the distribution of *agr* types between the infection and commensal groups. The *agr* system plays a major role in staphylococcal pathogenesis. According to a study conducted by Jarraud et al., different *agr* types are associated with different patterns of *S*. *aureus* diseases [16]. For example, *agr* type IV is associated with generalized exfoliative syndromes. Endocarditis strains mainly possess *agr* types I and II. However, in our study, we did not find any relationship between *agr* types and the patterns of *S*. *lugdunensis* disease. One possible reason is that our bacterial isolates were mostly from patients with bacteremia. No isolates were obtained from wound infections, and the number of isolates from other clinical samples was small. However, the *agr* types were highly correlated with the SCCmec types, probably because these isolates belonged to the same clones.

Most of our isolates were positive for δ-like hemolysin activity. Only 10% of the isolates were nonhemolytic. This result was consistent with that of a previous report [36]. A study revealed that the synergistic hemolytic activity of *S*. *lugdunensis* is distinct from that of *S*. *aureus* [37]. Unlike the δ-like hemolysin activity of *S*. *aureus*, which is controlled by the *agr-hld* system, that of *S*. *lugdunensis* is encoded by the *slush* locus, which belongs to the PSM family and is controlled by the *agr* locus. In animal models, the *agr*-defective mutants are attenuated for virulence [38]. However, in recent studies, *agr*-defective *S*. *aureus* was associated with persistent bacteremia and increased mortality [39, 40]. In our study, no difference was observed in the *agr* function between the infection and commensal groups. Considering the impact of *agr* functionality on staphylococcal pathogenesis, the role of *agr* in *S*. *lugdunensis* infection still requires further investigation.

Our study has several limitations: first, the isolates in our study were mostly obtained from blood culture. No isolates from wound or abscess culture were included. Second, most commensal isolates were obtained from the skin flora of patients and no isolates collected from healthy volunteers were available for further comparison. Third, the infectious isolates or commensals were classified on the basis of clinical criteria. Fourth, the data were collected from a single hospital, and the number of community-associated *S*. *lugdunensis* isolates was small.

In conclusion, the two most common SCCmec types carried by *S*. *lugdunensis* were types V and II. PFGE typing revealed the presence of five main clones (pulsotypes A, J, M, N, and P) at our hospital. Compared with the infection group, the commensal group showed a higher frequency of multiple drug resistance. By contrast, the commensal group showed higher genetic diversity, as evident in PFGE typing. The infectious isolates more frequently carried SCCmec type V, and the commensal groups showed a higher frequency of SCCmec type II. No difference was observed in *agr* types and in the presence of δ-hemolysin activity between the infection and commensal groups.

## Supporting Information

S1 Table Detailed information of 118 S. lugdunensis isolates (DOCX)
