# Supplementary material for: Molecular Characteristics of Disease-Causing and Commensal Staphylococcus lugdunensis Isolates from 2003 to 2013 at a Tertiary Hospital in Taiwan
Source: PLoS One. 2015 Aug 6;10(8):e0134859. doi: 10.1371/journal.pone.0134859 (PMC4527845; doi:10.1371/journal.pone.0134859)
Supplement: S1 Table — (DOCX) [file pone.0134859.s001.docx]

Table S1. Detailed information of 118 *S. lugdunensis* isolates

| Isolate number | Samples | Infectious foci | Penicillin | Oxacillin | Clindamycin | Erythromycin | TMP–SMX | Pulsotype | SCCmectype | *agr* type | Hemolysin activity |
| --- | --- | --- | --- | --- | --- | --- | --- | --- | --- | --- | --- |
| 1 | Blood | primary | Resistant | Sensitive | Sensitive | Sensitive | Sensitive | J |  | II | Positive |
| 2 | Blood | Intra-abdomen | Resistant | Sensitive | Resistant | Resistant | Sensitive | J |  | II | Positive |
| 3 | Blood | primary | Resistant | Sensitive | Resistant | Resistant | Sensitive | J |  | II | Positive |
| 4 | Blood | Catheter | Resistant | Sensitive | Sensitive | Sensitive | Sensitive | J |  | II | Positive |
| 6 | Blood | Commensals | Sensitive | Sensitive | Resistant | Resistant | Sensitive | Q |  | I | Positive |
| 8 | Blood | Arteriovenous graft/fistula | Sensitive | Sensitive | Sensitive | Resistant | Sensitive | P |  | I | Positive |
| 9 | Blood | primary | Resistant | Sensitive | Resistant | Resistant | Sensitive | L |  | I | Positive |
| 10 | Blood | primary | Sensitive | Sensitive | Sensitive | Sensitive | Sensitive | I |  | I | Positive |
| 11 | Blood | Infective endocarditis | Sensitive | Sensitive | Sensitive | Sensitive | Sensitive | I |  | I | Negative |
| 12 | Blood | primary | Sensitive | Sensitive | Sensitive | Sensitive | Sensitive | I |  | I | Positive |
| 14 | Blood | Arteriovenous graft/fistula | Resistant | Sensitive | Sensitive | Sensitive | Sensitive | Z |  | II | Positive |
| 15 | Blood | primary | Sensitive | Sensitive | Sensitive | Sensitive | Sensitive | F |  | I | Positive |
| 16 | Blood | Skin and soft tissue | Resistant | Sensitive | Sensitive | Resistant | Resistant | P |  | I | Positive |
| 18 | Blood | primary | Resistant | Sensitive | Sensitive | Sensitive | Sensitive | J |  | II | Negative |
| 19 | Blood | Arteriovenous graft/fistula | Resistant | Resistant | Sensitive | Sensitive | Sensitive | C | V | II | Positive |
| 20 | Blood | primary | Resistant | Resistant | Sensitive | Sensitive | Sensitive | A | V | II | Positive |
| 21 | Blood | Commensals | Resistant | Resistant | Sensitive | Sensitive | Sensitive | B | V | II | Positive |
| 22 | Blood | Commensals | Resistant | Resistant | Resistant | Resistant | Sensitive | N | II | I | Positive |
| 23 | Blood | Catheter | Sensitive | Sensitive | Sensitive | Sensitive | Sensitive | M |  | I | Positive |
| 24 | Blood | primary | Sensitive | Sensitive | Sensitive | Sensitive | Sensitive | M |  | II | Positive |
| 25 | Ascites | Intra-abdomen | Resistant | Sensitive | Sensitive | Sensitive | Sensitive | L |  | II | Positive |
| 26 | Blood | Bone and joints | Sensitive | Sensitive | Sensitive | Sensitive | Sensitive | M |  | I | Positive |
| 27 | Amniotic fluid | Genital system | Sensitive | Sensitive | Sensitive | Sensitive | Sensitive | M |  | I | Positive |
| 28 | Blood | Infective endocarditis | Sensitive | Sensitive | Resistant | Resistant | Sensitive | J |  | II | Positive |
| 29 | Blood | Commensals | Sensitive | Sensitive | Sensitive | Sensitive | Sensitive | J |  | II | Negative |
| 30 | Ascites | Commensals | Resistant | Sensitive | Sensitive | Sensitive | Sensitive | Z |  | II | Positive |
| 31 | Blood | Commensals | Resistant | Resistant | Resistant | Resistant | Sensitive | N | II | II | Negative |
| 32 | Blood | Commensals | Resistant | Resistant | Resistant | Resistant | Sensitive | M | II | I | Negative |
| 33 | Blood | Infective endocarditis | Sensitive | Sensitive | Sensitive | Sensitive | Sensitive | M |  | I | Positive |
| 34 | Blood | Arteriovenous graft/fistula | Resistant | Sensitive | Sensitive | Sensitive | Sensitive | AE |  | II | Positive |
| 35 | Blood | Commensals | Sensitive | Sensitive | Sensitive | Sensitive | Sensitive | H |  | II | Positive |
| 36 | Blood | Catheter | Resistant | Resistant | Resistant | Resistant | Sensitive | N | NT | I | Positive |
| 37 | Blood | Commensals | Resistant | Sensitive | Sensitive | Sensitive | Sensitive | J |  | II | Positive |
| 38 | Blood | Commensals | Resistant | Sensitive | Resistant | Resistant | Sensitive | W |  | I | Positive |
| 39 | Blood | primary | Sensitive | Sensitive | Resistant | Resistant | Sensitive | R |  | I | Positive |
| 42 | Blood | Commensals | Sensitive | Sensitive | Sensitive | Sensitive | Sensitive | C |  | II | Positive |
| 43 | Blood | Commensals | Resistant | Sensitive | Sensitive | Sensitive | Sensitive | AG |  | I | Positive |
| 44 | Blood | primary | Resistant | Resistant | Sensitive | Sensitive | Sensitive | A | V | II | Positive |
| 46 | Blood | Arteriovenous graft/fistula | Resistant | Sensitive | Sensitive | Sensitive | Sensitive | Z |  | II | Positive |
| 47 | Blood | primary | Resistant | Resistant | Sensitive | Sensitive | Sensitive | A | V | II | Positive |
| 48 | Blood | Commensals | Resistant | Resistant | Sensitive | Sensitive | Sensitive | AF | V | II | Positive |
| 49 | Blood | Infective endocarditis | Sensitive | Sensitive | Sensitive | Resistant | Sensitive | U |  | I | Positive |
| 51 | Blood | Commensals | Resistant | Resistant | Resistant | Resistant | Sensitive | N | II | I | Positive |
| 52 | Blood | primary | Resistant | Resistant | Resistant | Resistant | Sensitive | N | II | I | Positive |
| 53 | Blood | Intra-abdomen | Sensitive | Sensitive | Sensitive | Sensitive | Sensitive | F |  | I | Positive |
| 54 | Synovial fluid | Bone and joints | Resistant | Sensitive | Resistant | Resistant | Sensitive | J |  | I | Positive |
| 55 | Ascites | Intra-abdomen | Resistant | Resistant | Sensitive | Sensitive | Sensitive | A | V | II | Positive |
| 56 | Blood | primary | Resistant | Sensitive | Sensitive | Sensitive | Sensitive | X |  | II | Positive |
| 57 | Blood | primary | Resistant | Resistant | Sensitive | Sensitive | Sensitive | A | V | II | Positive |
| 59 | Blood | Commensals | Resistant | Resistant | Sensitive | Sensitive | Sensitive | A | V | II | Positive |
| 60 | Blood | Commensals | Sensitive | Sensitive | Sensitive | Sensitive | Sensitive | AC |  | II | Positive |
| 61 | Blood | Catheter | Resistant | Resistant | Sensitive | Sensitive | Sensitive | A | V | II | Positive |
| 62 | Blood | Commensals | Resistant | Sensitive | Resistant | Resistant | Sensitive | F |  | I | Positive |
| 63 | Blood | primary | Sensitive | Sensitive | Sensitive | Sensitive | Sensitive | Y |  | II | Negative |
| 64 | Blood | Commensals | Resistant | Sensitive | Sensitive | Sensitive | Sensitive | A |  | II | Positive |
| 65 | Blood | primary | Resistant | Sensitive | Sensitive | Sensitive | Sensitive | B |  | II | Positive |
| 66 | Blood | Commensals | Sensitive | Sensitive | Sensitive | Sensitive | Sensitive | AD |  | II | Positive |
| 68 | Blood | Commensals | Resistant | Sensitive | Sensitive | Sensitive | Sensitive | K |  | II | Positive |
| 69 | Ascites | Commensals | Resistant | Sensitive | Sensitive | Resistant | Sensitive | AA |  | II | Positive |
| 70 | Blood | Commensals | Resistant | Sensitive | Sensitive | Sensitive | Sensitive | A |  | II | Positive |
| 71 | Blood | Commensals | Resistant | Sensitive | Sensitive | Sensitive | Sensitive | A |  | II | Positive |
| 72 | Blood | Commensals | Resistant | Resistant | Sensitive | Sensitive | Sensitive | AB | V | II | Positive |
| 73 | Blood | Catheter | Resistant | Resistant | Sensitive | Sensitive | Sensitive | A | V | II | Positive |
| 74 | Blood | primary | Resistant | Sensitive | Sensitive | Sensitive | Sensitive | G |  | I | Positive |
| 75 | Blood | Catheter | Resistant | Resistant | Sensitive | Sensitive | Sensitive | A | V | II | Positive |
| 76 | Blood | Commensals | Resistant | Resistant | Sensitive | Sensitive | Sensitive | A | V | II | Negative |
| 77 | Blood | Commensals | Resistant | Resistant | Resistant | Resistant | Sensitive | N | II | I | Positive |
| 78 | Blood | Catheter | Resistant | Sensitive | Sensitive | Sensitive | Sensitive | Z |  | II | Negative |
| 79 | Blood | Commensals | Sensitive | Sensitive | Sensitive | Sensitive | Sensitive | J |  | II | Positive |
| 80 | Blood | Commensals | Resistant | Sensitive | Sensitive | Sensitive | Sensitive | AH |  | I | Positive |
| 81 | Blood | Bone and joints | Resistant | Sensitive | Sensitive | Resistant | Sensitive | P |  | I | Positive |
| 82 | Body fluid | Skin and soft tissue | Resistant | Resistant | Sensitive | Sensitive | Sensitive | A | V | II | Positive |
| 84 | Blood | Commensals | Sensitive | Sensitive | Resistant | Resistant | Sensitive | M |  | I | Positive |
| 85 | Blood | primary | Resistant | Resistant | Sensitive | Sensitive | Sensitive | A | V | II | Positive |
| 90 | Blood | Commensals | Resistant | Resistant | Resistant | Resistant | Sensitive | N | II | I | Positive |
| 91 | Pleural effusion | Lung | Resistant | Sensitive | Sensitive | Sensitive | Sensitive | J |  | II | Positive |
| 92 | Blood | primary | Resistant | Sensitive | Sensitive | Sensitive | Sensitive | S |  | I | Positive |
| 93 | Synovial fluid | Bone and joints | Resistant | Sensitive | Sensitive | Sensitive | Sensitive | K |  | II | Positive |
| 94 | Blood | primary | Resistant | Resistant | Sensitive | Sensitive | Sensitive | A | VT | II | Positive |
| 99 | Blood | Commensals | Resistant | Resistant | Sensitive | Sensitive | Sensitive | A | V | II | Positive |
| 100 | Blood | Commensals | Resistant | Resistant | Resistant | Resistant | Sensitive | O | II | I | Negative |
| 101 | Blood | Commensals | Resistant | Sensitive | Resistant | Resistant | Sensitive | F |  | I | Positive |
| 103 | Blood | Commensals | Resistant | Sensitive | Resistant | Resistant | Sensitive | P |  | I | Positive |
| 104 | Blood | primary | Resistant | Resistant | Resistant | Resistant | Sensitive | A | V | II | Positive |
| 106 | Body fluid | Skin and soft tissue | Resistant | Sensitive | Sensitive | Sensitive | Sensitive | H |  | I | Positive |
| 107 | Blood | Commensals | Resistant | Sensitive | Sensitive | Sensitive | Sensitive | B |  | II | Positive |
| 108 | Blood | Commensals | Resistant | Sensitive | Sensitive | Sensitive | Sensitive | T |  | I | Positive |
| 110 | Blood | Commensals | Resistant | Sensitive | Resistant | Resistant | Sensitive | P |  | I | Positive |
| 111 | Blood | Commensals | Sensitive | Sensitive | Sensitive | Sensitive | Sensitive | F |  | I | Positive |
| 112 | Blood | Commensals | Resistant | Resistant | Sensitive | Sensitive | Sensitive | A | V | II | Positive |
| 113 | Blood | Commensals | Resistant | Sensitive | Sensitive | Resistant | Sensitive | E |  | II | Positive |
| 114 | Ascites | CAPD peritonitis | Resistant | Sensitive | Sensitive | Sensitive | Sensitive | B |  | II | Negative |
| 115 | Blood | Catheter | Sensitive | Sensitive | Sensitive | Sensitive | Sensitive | P |  | I | Positive |
| 117 | Blood | Arteriovenous graft/fistula | Resistant | Sensitive | Sensitive | Sensitive | Sensitive | J |  | II | Positive |
| 118 | Blood | primary | Resistant | Resistant | Resistant | Resistant | Sensitive | N | II | I | Positive |
| 119 | Blood | Bone and joints | Resistant | Sensitive | Resistant | Resistant | Sensitive | H |  | II | Positive |
| 120 | Blood | primary | Resistant | Sensitive | Sensitive | Sensitive | Sensitive | N |  | I | Positive |
| 122 | Blood | primary | Resistant | Sensitive | Sensitive | Sensitive | Sensitive | A |  | II | Positive |
| 123 | Blood | primary | Resistant | Sensitive | Sensitive | Sensitive | Sensitive | J |  | II | Positive |
| 124 | Ascites | CAPD peritonitis | Sensitive | Sensitive | Sensitive | Sensitive | Sensitive | D |  | I | Positive |
| 125 | Blood | Commensals | Resistant | Resistant | Sensitive | Resistant | Resistant | P | IV | I | Positive |
| 126 | Blood | Commensals | Resistant | Resistant | Sensitive | Sensitive | Sensitive | C | V | II | Positive |
| 129 | Blood | Commensals | Resistant | Sensitive | Resistant | Resistant | Sensitive | V |  | I | Positive |
| 130 | Blood | Bone and joints | Sensitive | Sensitive | Sensitive | Sensitive | Sensitive | I |  | II | Positive |
| 131 | Blood | Catheter | Resistant | Resistant | Sensitive | Sensitive | Sensitive | A | V | II | Positive |
| 133 | Blood | Commensals | Resistant | Sensitive | Sensitive | Sensitive | Sensitive | D |  | I | Positive |
| 134 | Cerebrospinal fluid | Central nervous system | Resistant | Resistant | Sensitive | Sensitive | Sensitive | A | V | II | Positive |
| 135 | Blood | Commensals | Resistant | Resistant | Sensitive | Sensitive | Sensitive | A | VT | II | Negative |
| 136 | Blood | Commensals | Resistant | Resistant | Resistant | Resistant | Sensitive | N | II | I | Positive |
| 137 | Blood | Commensals | Resistant | Resistant | Resistant | Resistant | Sensitive | J | II | I | Positive |
| 138 | Blood | primary | Resistant | Resistant | Resistant | Resistant | Sensitive | A | IV | II | Negative |
| 139 | Blood | primary | Resistant | Resistant | Resistant | Resistant | Sensitive | N | II | I | Positive |
| 141 | Blood | Commensals | Resistant | Sensitive | Sensitive | Sensitive | Sensitive | I |  | I | Positive |
| 142 | Blood | Commensals | Resistant | Resistant | Resistant | Resistant | Sensitive | N | II | I | Positive |
| 143 | Blood | primary | Sensitive | Sensitive | Sensitive | Sensitive | Sensitive | R |  | I | Positive |
| 144 | Blood | Catheter | Resistant | Resistant | Sensitive | Sensitive | Sensitive | A | VT | II | Positive |
| 145 | Blood | Commensals | Resistant | Resistant | Resistant | Resistant | Sensitive | D | NT | I | Positive |
| 146 | Blood | Commensals | Resistant | Resistant | Resistant | Resistant | Sensitive | M | II | I | Positive |
